# Functional Connectivity Derived From Electroencephalogram in Pharmacoresistant Epileptic Encephalopathy Using Cannabidiol as Adjunctive Antiepileptic Therapy

**DOI:** 10.3389/fnbeh.2021.604207

**Published:** 2021-02-23

**Authors:** Lilia Maria Morales Chacón, Lidice Galan García, Sheyla Berrillo Batista, Judith González González, Abel Sánchez Coroneaux

**Affiliations:** ^1^Department of Clinical Neurophysiology/Video EEG Unit, International Center for Neurological Restoration, Havana, Cuba; ^2^Department of Neuroinformatics, Cuban Neuroscience Center, La Habana, Cuba

**Keywords:** epileptic encephalopathy, cannabidiol, functional connectivity, electroencephalogram, graph theory

## Abstract

To explore brain function using functional connectivity and network topology derived from electroencephalogram (EEG) in patients with pharmacoresistant epileptic encephalopathy with cannabidiol as adjunctive antiepileptic treatment. Sixteen epileptic patients participated in the study, six of whom had epileptic encephalopathy with a stable dose of cannabidiol Epidiolex (CBD) as adjunctive therapy. Functional connectivity derived from EEG was analyzed based on the synchronization likelihood (SL). The analysis also included reconstructing graph-theoretic measures from the synchronization matrix. Comparison of functional connectivity data between each pathological group with the control group was carried out using a nonparametric permutation test applied to SL values between pairs of electrodes for each frequency band. To compare the association patterns between graph-theoretical properties of each pathological group with the control group, *Z* Crawford was calculated as a measure of distance. There were differences between pairs of electrodes in all frequency bands evaluated in encephalopathy epileptic patients with CBD adjunctive therapy compared with the control (*p* < 0.05, permutation test). In the epileptic encephalopathy group without CBD therapy, the SL values were higher than in the control group for the beta, theta, and delta EEG frequency bands, and lower for the alpha frequency band. Interestingly, patients who had CBD as adjunctive therapy demonstrated greater synchronization for all frequency bands, showing less spatial distribution for alpha frequency compared with the control. When comparing both epileptic groups, those patients who had adjunctive CBD treatment also showed increased synchronization for all frequency bands. In epileptic encephalopathy with adjunctive CBD therapy, the pattern of differences for graph-theoretical measures according to *Z* Crawford indicated less segregation and greater integration suggesting a trend towards the random organization of the network principally for alpha and beta EEG bands. This exploratory study revealed a tendency to an overconnectivity with a random network topology mainly for fast EEG bands in epileptic encephalopathy patients using CBD adjunctive therapy. It can therefore be assumed that the CBD treatment could be related to inhibition of the transition of the interictal to ictal state and/or to the improvement of EEG organization and brain function.

## Introduction

There is little doubt about the therapeutic results of compounds derived from the cannabis Sativa plant in different medical conditions including epilepsy. Respecting pharmacoresistant epilepsies, arguments are based, among others, upon data about the importance of the endocannabinoid system in the onset and generation of seizures (Ludányi et al., 2008; Romigi et al., 2010; Goffin et al., 2011; Vaessen et al., 2012) together with multiple case reports indicating the anti-crisis effect of cannabis in patients with epilepsy (Consroe et al., 1975; Gordon and Devinsky, 2001; Mortati et al., 2007). Documentation from observational studies and randomized double-blind placebo-controlled trials (RCT) over the last decade point to the benefit in the treatment of seizures focusing on two epileptic encephalopathy conditions such as Dravet syndrome and Lennox-Gastaut Syndrome (LGS; Devinsky et al., 2016, 2017, 2018; Hausman-Kedem et al., 2018; Lattanzi et al., 2018, 2019).

Although studies suggest an overall positive effect on seizure control, little is known regarding the impact of cannabidiol (CBD) on electroencephalogram (EEG). Less proof exists to support the positive effects of cannabis on interictal epileptic activity in EEG (Hegde et al., 2012; McCoy et al., 2018) as the latest published research has not discussed in much detail EEG in patients using CBD.

On the other hand, the impact of cannabis on the brain of people with epilepsy using structural and functional neuroimaging have been poorly addressed, and have shown different patterns of alterations in structure and brain function at rest or while performing a cognitive task (Allendorfer and Szaflarski, 2017; Allendorfer et al., 2019). Some research findings have shown that interictal functional networks in epilepsy subjects may be characterized by increased connectivity (Douw et al., 2010a; Horstmann et al., 2010; Bartolomei et al., 2013, 2017; Clemens et al., 2013). Though the literature on interictal network topology using graph theory of epileptic patients is inconclusive, there is increasing empirical evidence for the hypothesis that changes in brain network topology might play a crucial role in epilepsy. There are also studies indicating that functional brain networks in epileptic patients differ from those of healthy subjects during the interictal stage. Furthermore, EEG networks analysis can be used to monitor the success of both pharmacological and non-pharmacological treatment (Clemens et al., 2014; Haneef and Chen, 2014) in pharmacoresistant epilepsy patients (Fraschini et al., 2014; Liang et al., 2018).

Given the growing interest in the use of CBD for pediatric pharmacoresistant epilepsy populations, it is therefore important to evaluate brain function in these patients. Considering that the EEG is an accurate cost-effective measure of both epileptic activity and of brain function, this article aims to explore functional connectivity and network topology derived from EEG in patients with pharmacoresistant epileptic encephalopathy using cannabidiol as adjunctive antiepileptic treatment.

## Materials and Methods

A total of 16 epilepsy patients participated in the study. Ten with epileptic encephalopathy, and six with epileptic encephalopathy using a stable dose of 25–50 mg/kg/day of cannabidiol Epidiolex as adjunctive therapy for 6–12 months. All patients had confirmed pharmacoresistant epilepsy failing at least three different anti-epileptic drugs (AEDs), along with an average of a minimum of four countable seizures per month. Age, gender, seizure duration, and number of AEDs were similar between both epileptic groups. The most frequent AEDs received in both groups were valproate and levetiracetam followed by clobazam. The use of clobazam was 50% in epileptic patients using CBD and 40% in non-used CBD *p* = 0.70 (the difference between two proportions). A healthy control group matched for age and gender was also studied, as shown in Table 1.

**Table 1:** Demographic and clinical data.

Variables	Epileptic encephalopathy with CBD *N* = 6	Epileptic encephalopathy without CBD *N* = 10	Control healthy group	*p*-value
Age*	13.6 ± 7.1 (5–23)	10.6 ± 3.2 (7–16)	15.37 ± 5.20 (9–24)	*p* = 0.32* $\chi_{\left(\text{2}\right)}^{\text{2}}$ = 2.26
Gender	*F* = 2 *M* = 4	*F* = 2 *M* = 8	*F* = 5 *M* = 5	
Concomitant AED*	3.1 ± 1.4 (2–5) 3 using Clobazan	2.7 ± 0.6 (2–4) 4 using Clobazan		*p* = 0.88** *U* = 31 *Z* adjusted = 0.21
Age at seizure onset*	6.4 ± 4.2 (4 month−11 years)	4.2 ± 3.1 (1 day–11 years)		*p* = 0.33** *U* = 13.5 *Z* adjusted = 0.95
Epilepsy duration*	6.4 ± 4.6 (2–15 years)	6.6 ± 2.6 (2–11)		*p* = 0.43** *U* = 18 *Z* adjusted = −0.78
Seizure frequency/month	15 Sz/day to 100 Sz/ day	8 Sz/day to 50 Sz/day		
Epilepsy type	LGS: 3 RE:1 HIE: 2	LGS: 3 RE:1 HIE: 2		
Epilepsy Etiology	*S* = 5 *U* = 1	*S* = 4 *I* = 3 *U* = 3		

*(mean ± SD) and range. AED, antiepileptic drugs, F, female; M, male; Sz, seizures; S, structural; U, unknown; I, infection; LGS, Lennox–Gastaut syndrome; RE, Rassmussen encephalitis; IHE, isquemic hipoxic encephalopathy; *Chi square χ^2^, **Mann-whitney U* (mean ± SD) and range.

Subjects were evaluated consecutively in the Video-EEG telemetry Unit of the International Center for Neurological Restoration, Havana, Cuba during 2018–2019. Diagnosis and classification of seizures and epilepsy were made using the 2017 ILAE Seizure Classification (Scheffer et al., 2017). Furthermore, a detailed electroclinical, neuroimmunological, as well as neuropsychological evaluation, was performed in all patients.

### EEG Acquisition and Analyses

Scalp EEG signals were recorded using the 10-20 international standard montage with a sampling frequency of 200 Hz. Additional extracranial electrodes were also utilized to gather EEG records from patients and controls. The reference electrode was positioned at FCz (an electrode placed between Fpz and Cz) and the ground electrode was located in Fpz.

EEG data acquisition was performed with a Medicaid-5 digital EEG system (Neuronic SA, Cuba), with 32 channels, a 256 Hz sampling rate, and a 16-bit analog-to-digital converter. Electrode Impedance was kept below 5 kΩ, and filters were set at 0.5 and 30 Hz with a 60 Hz notch filter. Hence, successive analyses were carried out only for delta, theta, alpha, and beta frequency bands.

During the eyes-closed resting state, EEG was recorded for at least 30 min. In the current study, a minimum 12 h- seizure-free period was assured. Also, special attention was given to keep the subjects awake with their eyes closed while recording. EEG segments with artifacts and drowsiness were excluded from the analyses in all evaluated groups. Further, EEG interpretation and analyses were done by two board-certified electroencephalographers (LM and SB).

### Functional Brain Connectivity Based on EEG

Functional connectivity was evaluated based on the synchronization likelihood (SL). Considering two EEG signals from different electrodes scalp locations as two simultaneously recorded time series E1 and E2, SL is defined as the conditional likelihood that E2 is in the same state at two different time points *i* and *j* given that E1 is in the same state at the same two times. Therefore SL denotes how well E1 and E2 are synchronized (coupled). SL values range between 1 (full synchronization) and values near 0 (low coupling or desynchronization. The values of the parameters used to estimate the SL were *l*_(lag)_ = 10, *m* = 2 (embedding dimension), w1 = *L* * *m* = 20 (Theiler correction for autocorrelation effects and should be at least of the order of the autocorrelation time; Theiler, 1986), w2 = Nt − w1 − 50 = 512 − 20 − 50 = 442 (is a window that sharpens the time resolution of the synchronization measure, and Nt = 512 time points or samples (maximum number of recurrences), Pref = 0.05 (preference probability). A full technical explanation of this measure and its features are presented in Stam et al. (2002).

In-house scripts developed at the Cuban Center for Neuroscience according to Stam et al. (2002) were used to compute the SL measure. SL computation was preceded by EEG raw signal filtering at different frequency bands [delta, δ (1–3.9 Hz), theta, θ (4–7.9 Hz) alpha, α (8–12.9 Hz), beta, β (13–29.9 Hz)].

For each subject, an SL functional matrix (C) of Ne × Ne (19 × 19)—where Ne is the electrode number—was obtained for each frequency-band and all time-windows. The resulting SL matrices were averaged across all epochs in each participant. Each element C*ij* of C matrix is the functional synchronization between EEG sensors “*i*” and “*j*” at a specific frequency band. The matrix C is symmetric, meaning that C*ij* = C*ji*, self-connections C*ii* were excluded, implying zeros in the diagonal of this symmetric matrix. Thus, we have Ne * (Ne − 1)/2 = 19 * 18/2 = 171 different functional SL values in C.

The analysis included:

- Reconstructing graph-theoretic measures.
- Calculating clustering coefficient.
- Calculating efficient measures (path length, global Efficiency).

### Graph-Theoretic Measures

Graph theoretical properties were reconstructed from the synchronization matrix and characterized by clustering coefficient (local connectedness measure), and the shortest path length (overall network integration measure). Likewise, global and local efficiency, as well as global connectivity, were evaluated.

The representation of the statistical association between distinct nodes was calculated using the following measures:

#### Clustering Coefficient

A cluster in a graph has come to be used to refer to a highly interconnected group of nodes. The fraction of existing edges between nodes adjacent to node *i*, over the maximum potential number of such edges, is known as the clustering coefficient C*i* of a node *i* (Watts and Strogatz, 1998). Thus, the clustering coefficient of the network C refers to the mean clustering coefficient among all network nodes. Further, a weighted clustering coefficient was used (Onnela et al., 2005) to measure network functional segregation.

#### Path Length

Path length can be described as the average shortest path length over all pairs of nodes in the network and is a measure of how efficient the information flow through the network is (Christodoulakis et al., 2014). The characteristic path length is well-delineated exclusively for connected pairs of nodes. To overcome this constraint, efficiency between a pair of nodes was defined as the inverse of the shortest distance between the nodes (Latora and Marchiori, 2001). The characteristic path length was also used to measure network functional integration.

The average shortest path length between all pairs of nodes in the network is defined as the characteristic path length of the network and is the most frequently used measure of functional integration (Watts and Strogatz, 1998).

#### Global Efficiency

Global efficiency is generally understood to mean a measure of functional integration and is the inverse of the mean shortest path length between each pair of nodes. It can be defined as the average efficiency over all pairs of nodes. Global and local efficiency can mirror the level of global and local information transfer of a graph, and are directly and effectively utilized to gauge the performance of a network (Latora and Marchiori, 2001).

### Statistical Analysis

A thorough comparison of functional connectivity data between each pathological group with the control group was carried out using a nonparametric permutation test.

In our study, we applied nonparametric permutation to SL values between pairs of electrodes for each frequency band. As the variables did not distribute normally (Shapiro–Wilk test, *p* < 0.05), the mean SL parameter between groups was compared using a nonparametric permutation test. The permutation test has several attractive features: (a) tests are distribution free controlling the experiment wise error for the simultaneous univariate comparisons; (b) no assumptions of an underlying correlation structure are needed; and (c) they offer exact *p*-values for any number of subjects, frequency bands and recording sites as long as the number of permutations is adequately high.

With the purpose of finding dissimilarities between groups in functional connectivity, we considered the SL parameter in which *p* pairs of electrodes and f frequency bands from SL functional connectivity matrix C were obtained for each subject.

Permutation methodology was based on the phases below:

The statistical difference between the two SL distributions was tested using permutation techniques. To make a choice for the global and marginal hypothesis, permutation *t* statistic corresponding to univariate hypothesis were combined. The *t* statistics combined were obtained from: (a) the maximum value of all the univariate statistics (max *t*); (b) maximum over the set of frequency bands for each pair of electrodes (max *t*_f_); (c) maximum over the set of pairs of electrodes at a particular frequency band (max *t*_s_).

In all cases, the use of *t*-max statistic distribution for each marginal hypothesis allowed precise significance thresholds corrected for the multiple comparisons along the dimension in which the maximum was taken. The procedure consists of the following stages:

Stage 1 Random permutation of the observations (SL) between the groups for 10,000 times. In each repetition, max *t*, max *t*_f,_ and max *t*_s_ were calculated.

Stage 2 Estimation of the empirical null distribution for the statistics calculated in the preceding stage.

Stage 3 Calculation of the *p-*value for the *t*-max statistics of the empirical null distribution.

Stage 4 Rejection of the null hypothesis (controlling the Type I error) for those *t*-max statistics of the original sample above the significance thresholds (i.e., *p*-value <0.05; Galán et al., 1997; Herrera-Díaz et al., 2016).

The effect size was computed using the Cohen’ *d* standardized measure (Cohen, 1988). The maximum effect size was calculated for an average over all channels.

According to Sawilowsky as a rule of thumb, this can be interpreted as follows: 0.5 as a “medium” effect, 0.8 as “large”, and 1.2 as “very large” (Sawilowsky, 2009).

Consequently, important connectivity (*p* < 0.05) in each frequency band was denoted on an *X/Y* coordinate based on the 10/20 system for each group individually. Further, results per group were represented over the scalp.

To compare the association patterns between graph-theoretical properties of each pathological group with the control group, *z* Crawford was calculated as a measure of distance.

The *z*-score that allows treating of the control sample statistics as sample statistics was described by Crawford and Howell (1998). The formula used by Crawford and Howell’s approach for a modified *t*-test is:

(1) $$t=\frac{X^{*}-\underline{X}}{S\sqrt{\frac{n+\text{1}}{n}}}$$

where X* is the patient’s score, $\underline{X}$ and *S* represent the mean and standard deviation of scores in the control sample, and n specifies the control sample size. The *p*-value obtained when the test is used tests significance, but it also indicates a point estimation of the abnormality of the patient’s score. Crawford and Howell’s procedure tests the null theory that a single patient does not belong to a control population. Also, Bonferroni’s correction was used to control the Family-wise Error Rate (FWER), which is the probability of having at least one false positive among the whole set of hypotheses considered as an alternative at the desired level α. In this work α =0.05 was used (Bonferroni’s correction = 0.0025).

## Results

### EEG Functional Connectivity

**Figure 1:**
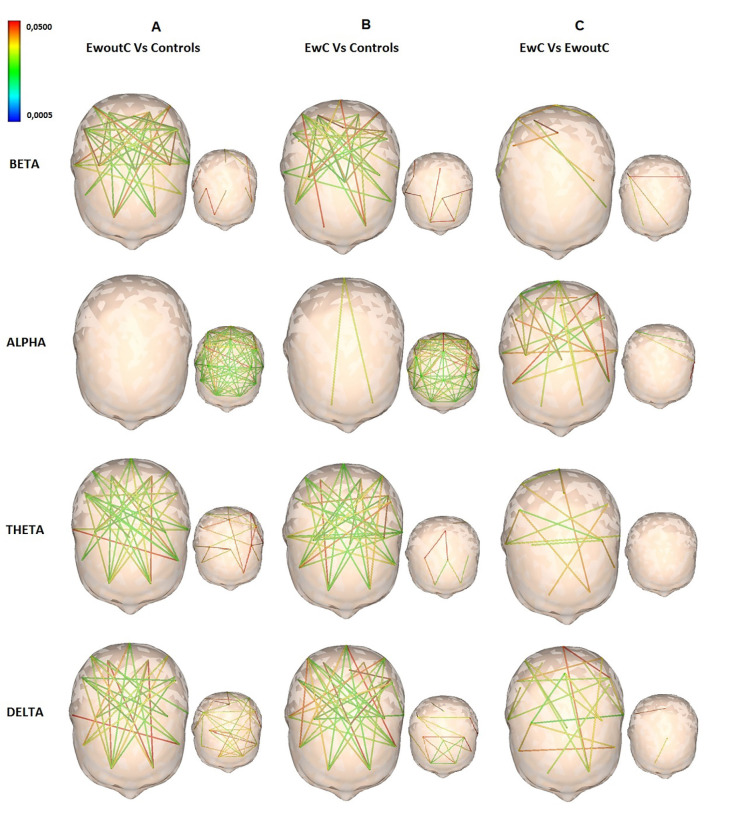
Illustrates pairs of electrodes with significant differences in synchronization likelihood (SL) values between patients and controls in beta, alpha, theta, and delta bands (patients > controls). The colored bar indicates the range of *p* values, in red representing 0.022 < *p* < 0.05, yellow 0.011 < *p* < 0.22, and blue *p* < 0.001. Differences were distributed over the entire scalp showing strong and significant electrodes SL couplings across all distances. Panel **(A)** shows an increase of SL in patients with epileptic encephalopathy without cannabidiol Epidiolex (CBD) adjunctive therapy for the beta, theta, and delta frequencies; and a decrease for alpha frequency compared with the control group. Panel **(B)** presents an increase of SL in patients with epileptic encephalopathy with CBD adjunctive therapy compared with the control group for the beta, alpha, theta, and delta frequencies showing less spatial distribution for alpha frequency. Panel **(C)** indicates increased synchronization for all frequency bands in patients with epileptic encephalopathy with adjunctive CBD when comparing both patient groups.

Functional connectivity based on the SL results indicated important differences (*p* < 0.05, permutation test), between pairs of electrodes in both groups of epileptic patients compared to the control. Figure 1 presents pairs of electrodes with significant SL values (*p* < 0.05) and effect sizes between groups in beta, alpha, theta, and delta bands. Differences were distributed over the whole scalp showing strong and meaningful electrode SL couplings across all distances. It can be observed from Figure 1A, that SL was higher in patients with epileptic encephalopathy without CBD than in the control group for beta, theta, and delta EEG frequency bands. However, the SL for the alpha frequency band was lower in relation to the control group. Significant differences *p* < 0.05, with medium and large effect sizes, were seen for beta (Cohen’ *d* = 0.5820) and alpha (Cohen’ *d* = 1.0388) EEG bands respectively. The effect sizes were small for slow-wave bands (for delta band Cohen’ *d* = 0.3975 and theta band Cohen’ *d* = 0.3270). As can be seen in Figure 1B, patients who had epileptic encephalopathy with CBD adjunctive therapy presented an increase of SL compared with the control group for the beta, alpha, theta, and delta frequencies, showing less spatial distribution for alpha frequency. Significant differences *p* < 0.05, with medium and large effect sizes, were also seen for beta (Cohen’ *d* = 0.4653) and alpha (Cohen’ *d* = 0.9524) bands, respectively. As shown in Figure 1C, when comparing both patient groups, subjects with epileptic encephalopathy with adjunctive CBD showed increased synchronization for slow and fast frequency bands. The effect sizes were small for all EEG except for the delta band (Cohen’ *d* = 0.4575, which was a medium effect. The effect sizes were 0.3148, 0.2319, and 0.2157 for theta, alpha, and beta bands, respectively.

### Graph Theory Based on EEG in Epileptic Encephalopathies With or Without Adjunctive Cannabidiol Therapy

In patients diagnosed with epileptic encephalopathy without adjunctive CBD treatment, differences with the control group were observed for deviations from graph theoretical measures according to *Z* Crawford in the alpha, beta, and delta frequency bands. As shown in Table 2, the difference direction represented in the mean of the *t* statistics indicated lower clustering coefficient and local efficiency, whereas the global efficiency was greater compared with the control group.

**Table 2:** Statistical differences (*t*-test) between graph theory measures of encephalopathy epileptic patients and healthy control sample based on the *z* individual value for each subject (Crawford’s approach).

Variables	Epileptic encephalopathy CBD group			Epileptic encephalopathy non-CBD group		
	Mean *t*-values	Statistics *t* (Ho: *μ* = 0; *df* = 4)	*p*-values	Mean *t*-values	Statistics *t* (Ho: *μ* = 0; *df* = 9)	*p*-values
Zcrawf-alpha clustering coefficient	4.00666	39.2971	0.000003	3.89590	8.6396	0.000006
Zcrawf-alpha path length	–1.12285	–4.9992	0.007494	–0.77795	–2.1810	0.054155
Zcrawf-alpha local efficiency	2.37207	22.1325	0.000025	2.47784	9.9902	0.000002
Zcrawf-alpha global efficiency	–2.59997	–16.0846	0.000087	–2.18080	–4.9629	0.000568
Zcrawf-alpha global connectivity	2.10682	22.6609	0.000022	1.72589	3.5383	0.005372
Zcrawf-beta clustering coefficient	1.08159	7.1742	0.001999	1.12795	9.6618	0.000002
Zcrawf-beta path length	0.13658	0.3684	0.731220	–0.63661	–5.0179	0.000523
Zcrawf-beta local efficiency	1.01745	6.9961	0.002197	0.98809	7.6680	0.000017
Zcrawf-beta global efficiency	–0.92687	–3.7821	0.019401	–1.29772	–24.4297	0.000000
Zcrawf-beta global connectivity	0.80529	3.1561	0.034313	0.90388	8.4076	0.000008
Zcrawf-theta clustering coefficient	0.39281	1.2676	0.273723	0.60178	3.2018	0.009463
Zcrawf-theta path length	1.20714	1.3802	0.239653	0.61625	1.4476	0.178346
Zcrawf-theta local efficiency	0.35845	1.0206	0.365166	0.53129	2.9284	0.015082
Zcrawf-theta global efficiency	0.00361	0.0092	0.993087	–0.33069	–1.1628	0.271918
Zcrawf-theta global connectivy	0.10068	0.3605	0.736678	0.45641	2.0628	0.066078
Zcrawf-delta clustering coefficient	0.82996	2.4170	0.072998	1.32238	15.6306	0.000000
Zcrawf-delta path length	0.76263	0.9942	0.376411	–0.94920	–3.3923	0.006859
Zcrawf-delta local efficiency	0.92826	2.5494	0.063346	1.33682	14.2008	0.000000
Zcrawf-delta global efficiency	–0.25476	–0.5400	0.617864	–1.17919	–10.1736	0.000001
Zcrawf-delta global connectivity	–0.39847	–1.0362	0.358629	0.47550	4.3368	0.001474

CBD group showed changes only in EEG beta and alpha frequency bands. The pattern of differences was characterized by lower clustering coefficient and local efficiency, as well as by an increase in global efficiency in patients with epileptic encephalopathy with adjunctive CBD therapy compared with the control group, which indicated less segregation and greater integration suggesting a trend towards the random organization of the network.

Most notably, subjects with adjunct CBD therapy showed less segregation and greater integration restricted to fast EEG frequencies (alpha and beta). However, patients with epileptic encephalopathy without CBD showed the same behavior concerning segregation and integration for both slow and fast frequency bands.

Table 2 provides the *t*-statistics to test differences in population means between graph theory measures of the epileptic patient samples and control sample based on the *z* individual value for each subject (Crawford’s approach). Respectively, the *p*-values indicate the probability for the differences between patients and controls; thus, providing a point estimate of the abnormality of the patient’s difference. Note that the variables with significant differences exhibited considerable deviations just in alpha and beta estimations of connectivity parameters in the CBD group. The non-CBD group also revealed anomalies in delta and theta parameters.

## Discussion

This study indicated functional brain network differences between epileptic encephalopathy using CBD adjunctive therapy and healthy control. In the epileptic encephalopathy group without CBD therapy, the difference direction revealed an increase in the synchronization for beta and theta frequency bands and a decrease for alpha frequency. However, patients with epileptic encephalopathy who used adjunctive CBD therapy showed greater synchronization during the interictal state for all EEG frequency bands than the control group, including alpha EEG band but with the lesser spatial distribution. The most consistent results were obtained for fast EEG frequency bands. A trend towards the random organization of the network (less segregation and greater integration) was also observed principally for fast EEG bands in the CBD epilepsy group.

There has been renewed academic interest in CBD therapy, in particular concerning the modulation of cortical excitability through the human endocannabinoid system. Several authors have revealed that increased connectivity is generally seen as a feature strongly related to interictal functional networks in epilepsy patients (Bartolomei et al., 2006, 2013; Bettus et al., 2008; Bosma et al., 2009; Douw et al., 2010a, b; Horstmann et al., 2010; van Dellen et al., 2012; Clemens et al., 2013).

Even though research on generalized epilepsy, explicitly, epileptic encephalopathy is very limited in the literature, our results are in agreement with other findings in genetic generalized epilepsy, which have shown a general increase in connectivity manifested in all aspects of network analyses (Ponten et al., 2009; Niso et al., 2015; Davis et al., 2019). In this study, an overconnectivity for all bands, except for the alpha band was also detected in patients with epileptic encephalopathy. Interestingly, those who used CBD therapy showed higher connectivity values for all bands including alpha in relation to those who were not given this therapy. This result could indicate a propensity for a better organization of brain electrical activity in these patients with CBD.

According to the literature reviewed so far, no studies exist on graph theory in epileptic patients using CBD as adjunctive therapy. More recently, graph theory research has been progressively used to analyze brain networks in different structural and functional modalities (Chiang and Haneef, 2014; Pedersen et al., 2015). It is important to notice that functional and structural connectivity clarifies not only that but also the extent to which different brain zones are connected whereas network analysis using graph theory provides a framework to characterize the topological organization of functional and structural networks.

The most common parameters utilized in neuronal network analyses using graph theory are the clustering coefficient and the characteristic path length. In this study, five global metrics were used to find a pattern with decreased clustering and increased global efficiency. The clustering coefficient allowed to define the local segregation property of the network, and it was used to assess the network capability to share specialized data while the path length and global efficiency were used to evaluate the capacity of the network as a whole for inner-exchange information. A short path length, a low clustering, and a high global efficiency/local efficiency generally represent a small-world topology of the network and characterizes an optimal organization for communication efficiency (Watts and Strogatz, 1998; Rubinov et al., 2009).

In the present study, the graph metric analysis indicated a lower clustering coefficient in patients with epileptic encephalopathy observed for the alpha, beta, and delta frequency bands compared with the control group while in epileptic encephalopathy patients with CBD treatment this organizational pattern was only observed in the EEG fast frequency bands (alpha and beta).

This study produced results that are partially in line with those of other studies in people diagnosed with generalized epilepsy (Ponten et al., 2009; Niso et al., 2015; Davis et al., 2019). In Davis PE, study graphs metric analysis indicates that children who developed epileptic spasms presented increased local and long-range EEG connectivity with less segregation of graph regions into distinct modules (Davis et al., 2019). Other authors have demonstrated, using surface EEG and MEG during generalized absence seizures, a potential association between a more regular network topology and seizure generation (Ponten et al., 2009; Gupta et al., 2011). A significant finding to emerge from this study is that the random network topology, principally for fast EEG frequency in patients using CBD, may represent an inhibition mechanism for the conversion of the interictal state to seizure.

Although there has been little agreement on whether traditional frequency bands are certainly fixed entities, each frequency band has been associated with different cognitive functions (Younus et al., 2018). Interpreting frequency-dependent network changes in epilepsy provides valuable information. Consequently, it has been argued that higher (10–13 Hz) and lower (8–10 Hz) alpha frequency band are involved in different cerebral processes (Klimesch, 1999; Uhlhaas and Singer, 2006). In this study, both SL and graphs metric analysis indicated that network analyses for all EEG frequency bands in epileptic encephalopathy patients with CBD were different compared with the control and with epileptic patients without CBD.

EEG connectivity in both epileptic groups showed greater synchronization and a trend towards the random organization of the network in contrasting ways with a dependent frequency pattern indicating the foremost variations for the high-frequency bands in patients using CBD. The similarity in the behavior of the epileptic groups accords with earlier observations in generalized epilepsy. The connectivity pattern observed in this study could be associated with the slowing of EEG activity in epileptic encephalopathy which is often related to brain dysfunction as well as to the side effects and neurotoxicity of many AED (Ponten et al., 2009; Niso et al., 2015; Davis et al., 2019) whereas the results in SL and graphs metric analysis, specifically for fast EEG band in epileptic patients treated with CBD could allude to an improvement in both EEG activity and connectivity pattern.

Some studies have suggested that low and high-frequency bands reflect different aspects of information processing. Large-scale integration is primarily processed in the lower frequencies while local activity evolves in higher frequency bands (von Stein and Sarnthein, 2000; Koenig et al., 2005).

The findings observed in this study in encephalopathy patients with CBD mirror those of Niso’s study in generalized epilepsy subjects in which epileptic encephalopathy patients with CBD adjunctive therapy showed greater efficiency and lower eccentricity than the control group for the high-frequency bands using magnetoencephalographic evaluation (Niso et al., 2015).

There is also evidence that augmentation in regularity has a distinguishing frequency pattern depending on the type of epilepsy, which can move from high-frequency bands in cases of generalized epilepsy to the low-frequency theta band in focal epilepsy patients (Chavez et al., 2010; Horstmann et al., 2010; Bartolomei et al., 2013).

However, more research needs to be undertaken to better recognize what the results obtained in different frequency bands mean. It can therefore be assumed that the CBD treatment could be related to inhibition of the transition of the interictal to ictal state and/or to the improvement of EEG organization and brain function.

Due to the lack of publications describing anti-epileptic drug effects, findings of graph-theoretic analyses in epilepsy have been insufficient (Horstmann et al., 2010; Chiang and Haneef, 2014). Graph theory has the added advantage of being particularly sensitive to variations in brain network structure (Bullmore and Bassett, 2011). Equally, it could provide a valuable methodology for examining the influence of antiepileptic drugs and medical cannabis on brain networks.

Although the current study is based on a small sample of participants, the findings open a window for the potential use of EEG network analytical approach to be used in clinical practice to assess the effect of CBD as adjunctive therapy to treat pharmacoresistant epileptic encephalopathies.

## Data Availability Statement

The raw data supporting the conclusions of this article will be made available by the authors, without undue reservation.

## Ethics Statement

The studies involving human participants were reviewed and approved by International Center for Neurological Restoration. Written informed consent to participate in this study was provided by the participants’ legal guardian/next of kin.

## Author Contributions

LM designed the study and organized the manuscript. LG participated in the statistical analysis of the results and organization of the manuscript. SB carried out the EEG connectivity analysis. JG identified and evaluated the patients with epilepsy. AS participated in the patients’ evaluation. All authors contributed to the article and approved the submitted version.

## Conflict of Interest

The authors declare that the research was conducted in the absence of any commercial or financial relationships that could be construed as a potential conflict of interest.
